# Lnc GNG12-AS1 knockdown suppresses glioma progression through the AKT/GSK-3β/β-catenin pathway

**DOI:** 10.1042/BSR20201578

**Published:** 2020-08-18

**Authors:** Zijin Xiang, Qiaoli Lv, Xueru Chen, Xiuting Zhu, Shikun Liu, Dangchi Li, Xiangdong Peng

**Affiliations:** 1 Department of Pharmacy, The Third Xiangya Hospital, Central South University, Changsha, 410013, Hunan, China; 2 Jiangxi Key Laboratory of Translational Cancer Research, Jiangxi Cancer Hospital of Nanchang University, Nanchang, Jiangxi, China; 3 Department of Gynaecology, The Third Xiangya Hospital, Central South University, Changsha, 410013, Hunan, China; 4 Jiangxi University of Technology High School, Nanchang, Jiangxi, China

**Keywords:** β-catenin, AKT, glioma, GNG12-AS1

## Abstract

**Background:** Long non-coding RNAs (lncRNAs) are increasingly being regarded as regulators of glioma development. Notably, some studies report that GNG12-AS1 plays important functions and molecular mechanism in breast cancer, but there are no existing studies in glioma.

**Objective:** To analyze the biological functions and potential mechanisms of GNG12-AS1 in glioma.

**Methods:** We detected the expression of GNG12-AS1 in glioma tissues through analyzing TCGA data as well as our clinical samples. We then evaluated cell proliferation through MTT assay and colony formation and cell migration by transwell assay, wound healing assay and single cell tracking assay. After, we analyzed the effects of the AKT/GSK-3β/β-catenin through Western blotting and utilized the β-catenin agonist SKL2001 for the rescue experiment.

**Results:** GNG12-AS1 was highly expressed in glioma tissues. The silence of GNG12-AS1 inhibited the proliferation, migration and epithelial–mesenchymal transition of glioma cells, and reduced the activity of the AKT/GSK-3β/β-catenin pathway. Notably, SKL2001 could reverse cell migration as well as β-catenin expression in glioma cells with lower GNG12-AS1 expression.

**Conclusions:** GNG12-AS1 regulates proliferation and migration of glioma cells through the AKT/GSK-3β/β-catenin signaling and can perhaps be a new target for the treatment of glioma.

## Introduction

Glioma is one of the most common primary brain tumors in adults and has a 5-year survival rate of approximately 5% [1]. Although various treatments are available for glioma patients, including surgical resection, chemotherapy and radiation, the prognosis of glioma patients is poor [2]. Glioma can undergo highly invasive growth, which makes the tumor extremely prone to recurrence [3]. Based on such factors, it is necessary to identify the molecular mechanisms of glioma occurrence and development, and to discover specific biomarkers and therapeutic targets for glioma.

Long non-coding RNAs (lncRNAs) are defined as more than 200 nucleotide RNAs that lack protein-coding potential [4]. There is mounting evidence that lncRNA genes are similar to protein coding genes in some key respects, and they have realized important molecular functions in many cellular pathways and processes [5]. LncRNAs participate in the p53 regulatory network, interact with miRNAs or work as EMT regulators, etc [5–7]. For example, lncRNA PiHL regulates the stability of p53 protein in colorectal cancer through GRWD1/RPL11/MDM2 axis [8]. Meanwhile, HOTTIPEMT participates in EMT by activating Wnt/-catenin signaling [9].

Many abnormally expressed lncRNAs have been found to affect the progression of glioma [10]. LncRNA GNG12-AS1 is a novel lncRNA transcribed in an antisense orientation to the tumor suppressor DIRAS3 on chromosome 1 [11,12]. It is worth noting that an increasing number of studies have reported that GNG12-AS1 and DIRAS3 are synergistically down-regulated in breast cancer. The siRNA silence of GNG12-AS1 leads to transcriptional interference and DIRAS3 up-regulation, all of which impair the cell cycle and cell migration [11,12]. However, the role of GNG12-AS1 in glioma is still unclear.

The purpose of our study is to analyze the functions and mechanisms of GNG12-AS1 in glioma cells. We found that lnc GNG12-AS1 can affect the proliferation and migration of glioma cells. For further mechanism discovery, the silence of GNG12-AS1 inhibited the AKT/GSK-3β/β-catenin pathway.

## Materials and methods

### Clinical samples

Ninety-five glioma tissues and 20 human normal brain tissues that were acquired between the period of November 2010 and June 2013 from the Department of Neurosurgery, First Affiliated Hospital, Nanchang University (Nanchang, China) were used in the study. The glioma tissues were examined and verified by at least two experienced pathologists. All samples were immediately stored in liquid nitrogen after surgical removal and were then placed at −80°C for future experiments. The present study was approved by the Research Ethics Committee of First Affiliated Hospital of Nanchang University. All patients received informed consent.

### Cell cultures

T98G and HS683 cells were obtained from the American Type Culture Collection. All cells were maintained in Dulbecco’s modified Eagle’s medium (DMEM; Life Technologies, U.S.A.) of high sugar supplemented with 8% fetal bovine serum (FBS; Gibco) and 1% penicillin–streptomycin (Gibco). Cells cultured in moderate medium remained in a humid environment with a temperature of 37°C and 5% CO_2_.

### Cell transfection

Small interfering RNAs were purchased from Ribobio (Guangzhou, China) and the siRNA sequence of lnc GNG12-AS1 was 5′-GGATCTACGTGGCAAACAA-3′. The transfection of siRNA was performed using the reagent kit from Ribobio (Guangzhou, China) and the operation method was based on the kit instructions. For better experimental efficiency, cells, 48 h after transfection were used for the efficiency of detection and mechanism studies.

### Quantitative real-time PCR

TRIzol reagent (Invitrogen, Carlsbad, CA, U.S.A.) was utilized to extract total RNA from cells and tissues. The test method was performed according to the instructions, and non-enzyme equipment was used throughout the test. RNA was used for reverse transcription into cDNA by using Thermo Scientific RevertAid First Strand cDNA Synthesis Kit (Thermo Fisher Scientific, Wilmington, DE, U.S.A.). Next, three parallel duplicates in each group were set for quantitative real-time PCR (qRT-PCR) with the SYBR Green real-time PCR Kit (Takara, Dalian, China). The qPCR primer sequences were listed as follows: GNG12-AS1 forward 5′-CAGTTGGGCAAAGTTTCACTCC-3′ and reverse 5′-GCTCTGTGTTCTCATCCATATCTCA-3′; GAPDH forward 5′-TGACTTCAACAGCGACACCCA-3′ and reverse 5′-CACCCTGTTGCTGTAGCCAAA-3′.

### MTT assay

Cells were transfected for 24 h and seeded in a 96-well plate with a density of 3000 T98G cells/well and 2000 HS683 cells/well in 200-μl medium. At the specified point in time, 100 μl culture medium containing 20 μl MTT was added into each well and then the 96-well plates were placed in the incubator. After 4 h, 150 ul/well formanzan solution was used to dissolve the blue-purple crystals. The OD value of each well was detected at the absorbance of 490 nm.

### Colony formation

T98G and HS683 cells were transfected in a 24-well plate for 24 h, and 1000 cells/well were stored in a six-well plate. The medium was replaced every 4 days, and was discarded after 2 weeks. Then the six-well plate was gently washed with phosphate-buffered saline (PBS), fixed with methanol for 1 h and stained by 0.1% Crystal Violet for 1 h. Finally, the colonies were left after being washed through PBS and were photographed.

### Transwell assay

After 48 h of transfection, cells were digested from 12-well plates. A total of 200 μl FBS-free medium with 10000 cells was added to the upper compartment of the transwell chamber, while 500 μl medium with 10% FBS was added to the lower compartment. After the plates were cultured in the incubator for 6 h, the chambers were taken out, fixed with methanol and stained with 0.1% Crystal Violet. The chambers were then placed under a microscope for imaging. For the SKL2001 activated group, the agonist was added to the serum-free medium to activate the cells.

### Wound healing assay

When transfected T98G and HS683 cells grew to more than 90% in the 12-well plate, the 200-μl tips perpendicular to the marker lines were used to create the wound. After the cell fragments were washed by adding PBS, the cells were cultured in 2% FBS medium and the 12-well plate was placed under the microscope to take photos. The cell migration pictures were taken at a fixed position 24 h later.

### Single cell tracking assay

The Operetta High Content Imaging System is a mature technology which combines fully automatic high throughput fluorescence microscopy and multi-parameter quantitative image analysis, which provides researchers with quick and effective tools to quickly obtain specific influencing factors or effects of drugs in cells, such as cell migration, cell cycle and apoptosis, etc [13,14]. The transfected cells were seeded with 2000 cells per well in a 96-well plate of type 3599 and cultured in an Operetta High Content Imaging System at 37°C and 5% CO_2_ for 12 h. In the Operetta® High Content Imaging System, digital phase contrast images were acquired by every 1 h, and cell displacement of 12 h was compared by analyzing the Mean Square Displacement in the Harmony® High Content Imaging and Analysis Software.

### Western blotting

The total protein content of the cells was extracted by using RIPA buffer which contained 2% phosphatase inhibitor and 1% PMSF. A total of 100 μl protein buffer could be received for each hole of the six-well plate. Protein samples were separated by standard sodium dodecyl sulfate/lyacrylamide gel electrophoresis and transferred to the PVDF membranes. The membrane was soaked in 5% skimmed milk with TBST and sealed for 1 h at room temperature and was later incubated with the primary antibodies overnight at 4°C. The next day, the membranes were incubated with secondary antibodies and imprinted. Anti-GAPDH was diluted to 1:5000, while anti-N-cadherin, anti-PAKT, anti-vimentin, anti-AKT, anti-GSK-3β, anti-p-GSK-3β, anti-β-catenin, anti-slug and anti-E-cadherin were diluted to 1:2000. HRP-linked anti-human IgG was the secondary antibody and diluted to 1:10000. All antibodies were purchased from CST Biotechnology (Boston, MA, USA.). .and diluted with TBST.

### Statistical analysis

GraphPad Prism 8 was used for data statistical analysis. The two-tailed Student’s *t* test was used for intergroup analysis. The *P*-value less than 0.05 was considered statistically significant.

## Results

### The expression level of GNG12-AS1 was significantly increased in glioma tissues

**Figure 1:**
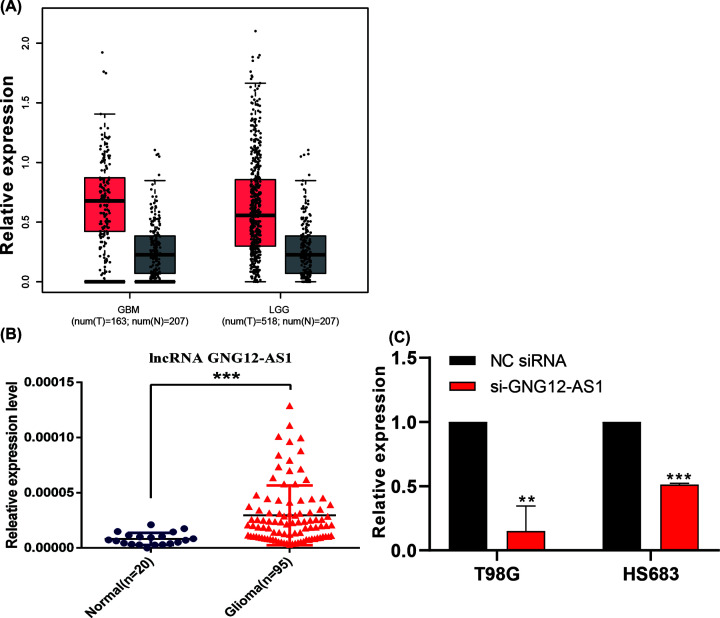
The expression of GNG12-AS1 in glioma tissues (**A**) The GNG12-AS1 expression in GBM and LGG by TCGA analysis. Gray represents normal tissue and red represents tumor tissue. (**B**) RT-PCR assay detected the GNG12-AS1 expression in 95 glioma tissues and 20 normal brain tissues. (**C**) RT-PCR assay examined the GNG12-AS1 expression in 48 h after transfection. **P<0.01, ***P<0.001.

First, we analyzed the expression of GNG12-AS1 in glioblastoma and low-grade glioma on the online analysis website GEPIA (http://gepia.cancer-pku.cn/index.html) [15] which combines matched TCGA and GTEx data. The analysis results illustrated that GNG12-AS1 was highly expressed in tumor tissues compared with normal tissues, whether in glioblastoma or low-grade glioma (Figure 1A). Then we extracted RNA from 95 glioma tumor tissues and 20 normal brain tissues and determined GNG12-AS1 expression in the tissues by RT-PCR. Similar to before, lncRNA was significantly higher in tumor tissues (Figure 1B). After transfection with siRNA, mRNA expression of GNG12-AS1 decreased (Figure 1C).

### GNG12-AS1 enhanced the proliferation of glioma cells

**Figure 2:**
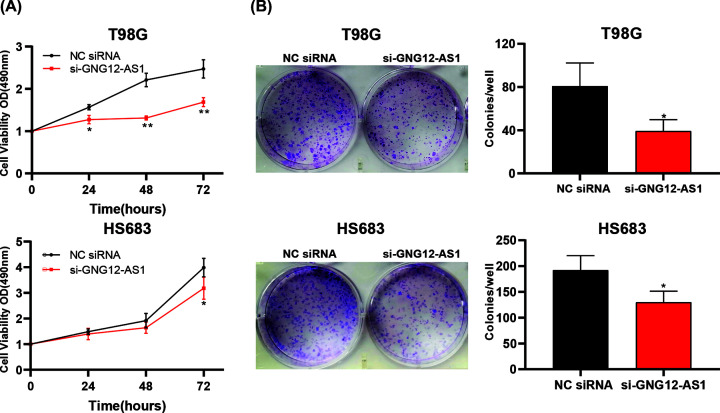
The effect of GNG12-AS1 knockdown on cell proliferation (**A**) MTT assay illustrated that the proliferation of T98G and HS683 cells transfected with si-GNG12-AS1 decreased compared with cells transfected with NC siRNA. (**B**) Colony formation assay demonstrated that GNG12-AS1 knockdown reduced the number of colonies in T98G and HS683 cells. *P<0.05, **P<0.01.

The MTT assay was used to detect the effect of GNG12-AS1 on the proliferation of glioma cells. The result showed that the knockdown of GNG12-AS1 in glioma cells led to a significant decrease in cell proliferation (Figure 2A). Moreover, GNG12-AS1 siRNA-transfected cells performed poorly in cell colony formation (Figure 2B).

### GNG12-AS1 promoted migration of glioma cells

**Figure 3:**
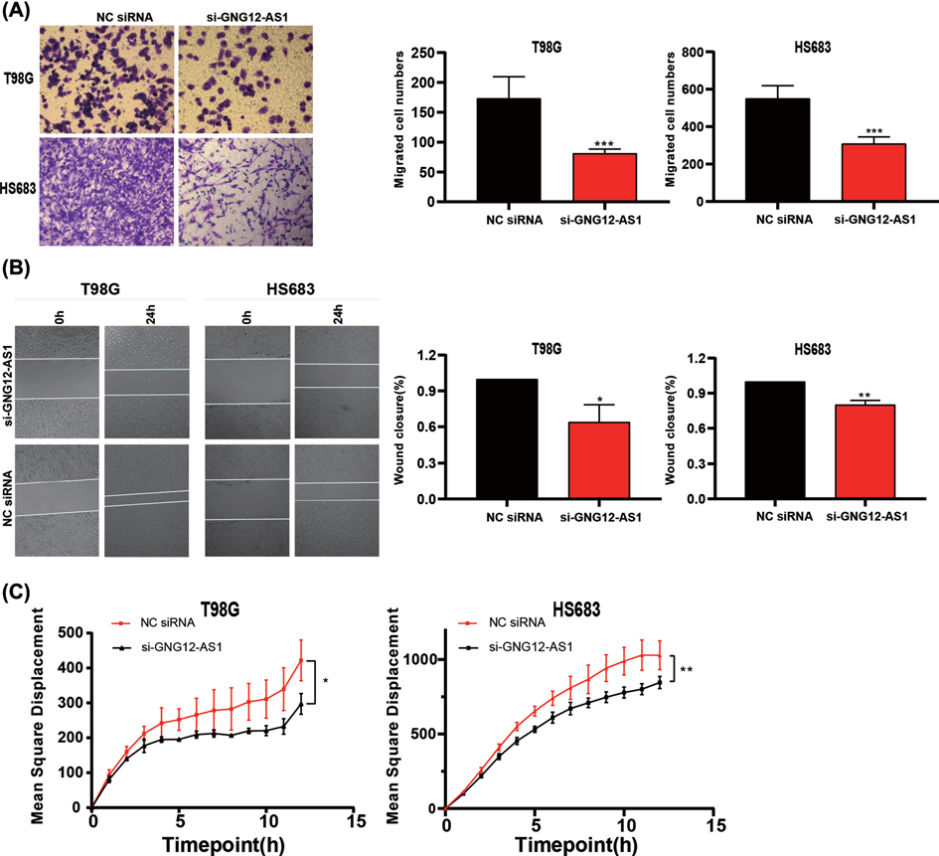
The influence of GNG12-AS1 knockdown on cell migration ability (**A**) The transwell migration assay indicated that it was difficult for T98G and HS683 cells with the silence of si-GNG12-AS1 to get through the chamber. (**B**) The wound healing experiment showed that GNG12-AS1 knockdown in T98G and HS683 cells significantly inhibited cell migration compared with the NC siRNA group. (**C**) For single cell tracking assay, the displacement of all individual cell migration was shorter in si-GNG12-AS1 knockdown cells. *P<0.05, **P<0.01, ***P<0.001.

Since cell metastasis is an important cause of glioma metastasis, three experiments were used to evaluate the effect of GNG12-AS1 on cell migration. Transwell assay results demonstrated that the amount of cells migration after GNG12-AS1 knockdown significantly decreased in the chambers (Figure 3A). Wound-healing experiments confirmed that the silence of GNG12-AS1 inhibited cell migration (Figure 3B). Furthermore, in order to observe the tracking of individual cells, we applied the Operetta High Content Imaging System to analyze the total mean square displacements of 12 h. As the results indicated, cells with low expression of GNG12-AS1 migrated more slowly (Figure 3C).

### GNG12-AS1 activated AKT/GSK-3β/β-catenin signaling pathway

**Figure 4:**
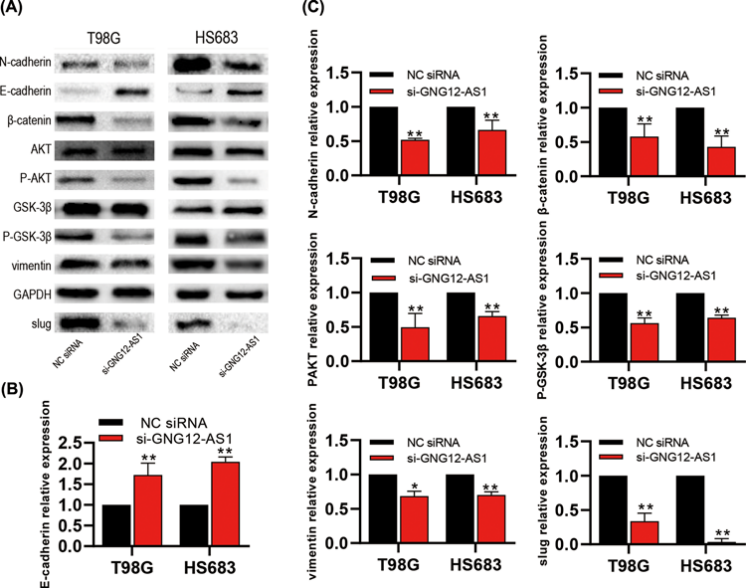
GNG12-AS1 down-regulation inhibited the AKT/GSK-3β/β-catenin signaling pathway in glioma cells (**A**–**C**) The Western blotting assay exhibited the expression level of EMT-related markers and pathway markers in si-GNG12-AS1 group and NC siRNA group. *P<0.05, **P<0.01.

As the above experimental results demonstrated, GNG12-AS1 had significant influence on cell migration. To further clarify the relevant mechanism of GNG12-AS1 on migration, we examined biomarkers of epithelial–mesenchymal transition, such as vimentin, N-cadherin, E-cadherin, slug. As expected, the results illustrated that the expression of these biomarkers was decreased after the GNG12-AS1 knockdown (Figure 4A,C), except that E-cadherin expression was increased (Figure 4B). Currently, some genes have been reported to modulate the EMT process in tumors through the AKT/GSK-3β/β-catenin signaling pathway [16,17]. Because of this, we also analyzed the effect of GNG12-AS1 on the pathway. The results reflected that GNG12-AS1 knockdown reduced the proteins expression of P-AKT, P-GSK-3β and β-catenin, and had no effect on total AKT and GSK-3β protein levels (Figure 4A,C). These results implied one possibility that GNG12-AS1 may regulate epithelial–mesenchymal transformation through the AKT/GSK-3β/β-catenin pathway to promote cell migration.

### β-catenin agonist reversed the effect of knocking down GNG12-AS1

**Figure 5:**
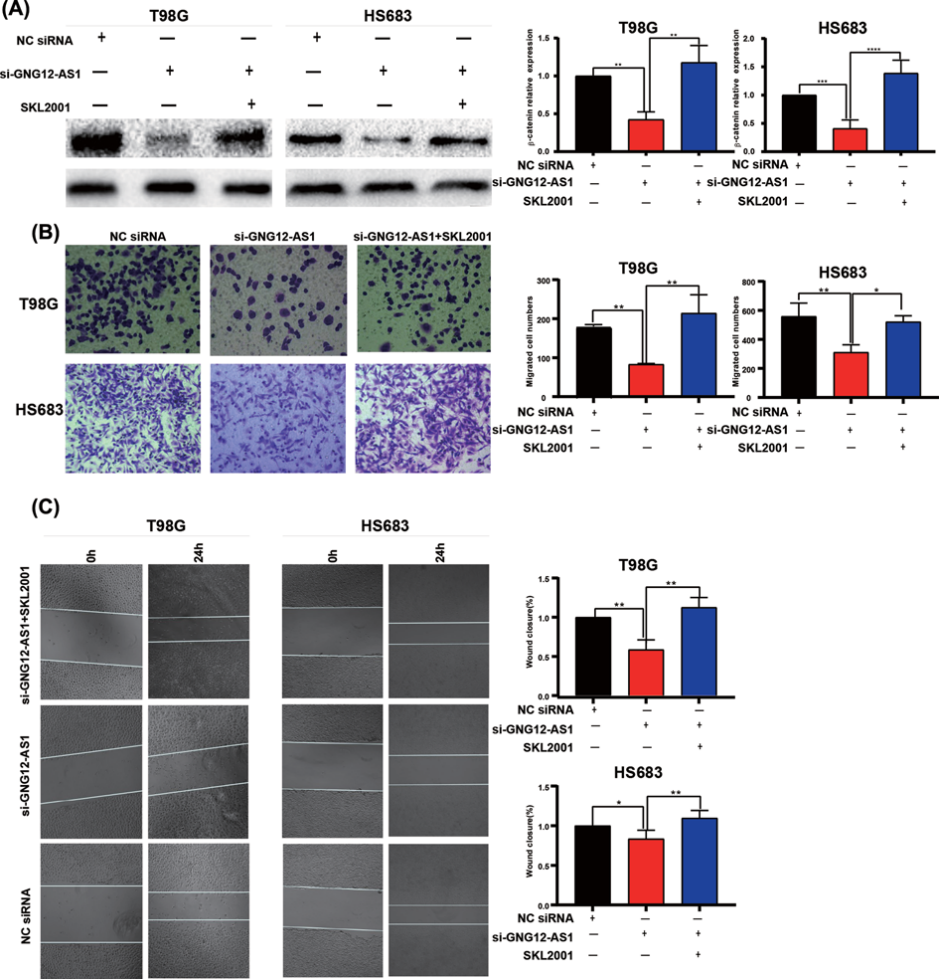
The effect of SKL2001 in si-GNG12-AS1 cells was analyzed (**A**) The Western blotting assay indicated that the protein level of β-catenin increased due to the addition of SKL2001. (**B**,**C**) The wound healing experiment and the transwell assay confirmed that the cell migration ability improved in the group of SKL2001 addition compared with the si-GNG12-AS1 group. *P<0.05, **P<0.01, ***P<0.001.

To further verify whether GNG12-AS1 promoted cell migration through the AKT/GSK-3β/β-catenin pathway, we treated the cells of GNG12-AS1 knockdown with β-catenin agonist SKL2001 (5 nm, 30 min) [18]. After SKL2001 activated the β-catenin pathway, the protein level of β-catenin was elevated (Figure 5A). Transwell assay results illustrated that cell migration was reversed after β-catenin activation by SKL2001, compared with GNG12-AS1 knockdown alone (Figure 5B). The wound-healing results also demonstrated that SKL2001 treatment restored the inhibition of migration after GNG12-AS1 knockdown (Figure 5C). In summary, our results suggest that GNG12-AS1 induces cell migration through the AKT/GSK-3β/β-catenin signaling pathway.

## Discussion

LncRNAs play significant roles in glioma progression, such as HOTAIR [19,20], ZEB1-AS1 [21], etc. Notably, previous studies have indicated that a novel lncRNA, lnc GNG12-AS1, is highly expressed in the pancreas, small intestine, testes, liver, breasts, cervix and adipose tissue. The primary function of GNG12-AS1 has been studied primarily in breast cancer [11,12]. In this study, we discovered that the expression of GNG12-AS1 was higher in glioma tissues than in normal tissues. Moreover, T98G and HS683 cells transfected with si-GNG12-AS1 have poor proliferation and migration ability. These results suggested that lnc GNG12-AS1 regulated glioma progression as an oncogene. Although it is interesting that GNG12-AS1 plays a role as a suppressor gene in breast cancer and an oncogene in glioma, some studies have shown that other lncRNAs have similar effect in human cancer. For instance, lnc MALAT1 has been reported to promote proliferation and inhibit apoptosis through derepressing Rap1B by sponging miR-101 in glioma cells [22], while it is also confirmed to inhibit breast cancer metastasis as a metastasis suppressor [23]. Consequently, the functional differences of GNG12-AS1 in glioma and breast cancer may indicate that the genes have different mechanisms in different tumors, which is worthy of further study.

Numerous researches illustrate that the occurrence of glioma is inseparable from the changes of signaling pathways [24,25]. Moreover, changes in the expression of lnc RNAs can cause abnormal expression of related pathways. For instance, Foxd2-as1/miR-185-5P/HMGA2 axis influences the PI3K/Akt signaling pathway to promote tumorigenesis and progression of glioma [26]. As a result, it may be hopeful for glioma treatment to explore potential molecular markers. Additionally, there is an abundance of research evidence that epithelialmesenchymal transformation has occurred in most tumors [27]. We found that vimentin, N-cadherin and slug expressions were decreased and E-cadherin expressions were increased in the low-expression GNG12-AS1 cells. Among the various signaling pathways acting on EMT, the Akt signaling pathway and the Wnt/β-catenin signaling pathway activate a variety of effector molecules [28]. Notably, the previous studies illustrated that Nobiletin and SCD1 regulate EMT of glioma cells via the Akt/GSK3β/β-catenin signaling axis [29,30]. Meanwhile, phosphorylation of AKT activates a variety of downstream protein substrates, including GSK-3β [31,32]. Specifically, GSK-3β is the degradation factor of β-catenin and a key factor of the Wnt/β-catenin signaling pathway, which can directly regulate the stability of β-catenin protein in the cytoplasm [33]. As a result, with the important role of AKT/GSK-3β/β-catenin pathway in EMT, we confirmed that the pathway was inhibited by GNG12-AS1 knockdown. The rescue experiments depicted that the effects of GNG12-AS1 were reversed after the activation of β-catenin. Because of the role of lncRNA at transcriptional and post-transcriptional levels, GNG12-AS1 may regulate the transcription or translation ability of some genes which influence the AKT/GSK-3β/β-catenin pathway in glioma cells. Further experiments need to be carried out.

In summary, we were the first to investigate the biological role of lncRNA GNG12-AS1 in glioma and found that GNG12-AS1 enhanced the proliferation and migration of glioma cells. Our study demonstrated that GNG12-AS1 promoted proliferation and migration of glioma cells through activating the AKT/GSK-3β/β-catenin signaling pathway. The results above suggested that the study of GNG12-AS1 could provide a new focus for the research of the occurrence and development of glioma.

## Abbreviations

DIRAS3: DIRAS family GTPase 3
EMT: Epithelial-mesenchymal transition
FBS: fetal bovine serum
Foxd2-as1: FOXD2 adjacent opposite strand RNA 1
GAPDH: glyceraldehyde-3-phosphate dehydrogenase
GRWD1: glutamate rich WD repeat containing 1
GSK-3β: glycogen synthase kinase 3 beta
HMGA2: high mobility group AT-hook 2
HOTAIR: HOX transcript antisense RNA
HRP: Horseradish Peroxidase
lncGNG12-AS1: long non-coding RNA GNG12, DIRAS3 and WLS antisense RNA 1
lncRNA: long non-coding RNA
MALAT1: metastasis associated lung adenocarcinoma transcript 1
MDM2: MDM2 proto-oncogene
MTT: 3-（4,5-dimethyl-2-thiazolyl）-2,5-diphenyl-2-H-tetrazolium bromide
OD: optical density
PBS: phosphate-buffered saline
piHL: p53 inhibiting lncRNA
qPCR: Real-time Quantitative PCR Detecting System
Rap1B: RAP1B, member of RAS oncogene family
RPL11: ribosomal protein L11
TBST: tris buffered saline containing 0.1% (v/v) Tween 20
TCGA: The Cancer Genome Atlas
